# Premature Expression of Foxp3 in Double-Negative Thymocytes

**DOI:** 10.1371/journal.pone.0127038

**Published:** 2015-05-15

**Authors:** Melanie M. Barra, David M. Richards, Ann-Cathrin Hofer, Michael Delacher, Markus Feuerer

**Affiliations:** Immune Tolerance, Tumor Immunology Program, German Cancer Research Center (DKFZ), Im Neuenheimer Feld 280, 69120 Heidelberg, Germany; Institut Pasteur, FRANCE

## Abstract

Peripheral immune regulation depends on the generation of thymic-derived regulatory T (tT_reg_) cells to maintain self-tolerance and to counterbalance overshooting immune responses. The expression of the T_reg_ lineage defining transcription factor Foxp3 in developing tT_reg_ cells depends on TCR signaling during the thymic selection process of these T cells. In this study, we surprisingly identify Foxp3^+^ immature thymocytes at the double-negative (DN) stage in transcription factor 7 (Tcf7)-deficient mice. These Foxp3^+^ cells did not express a TCR (β or γδ chains), CD3 or CD5 and therefore these cells were true DN cells. Further investigation of this phenomenon in a transgenic TCR model showed that Foxp3-expressing DN cells could not respond to TCR stimulation *in vivo*. These data suggest that Foxp3 expression in these DN cells occurred independently of TCR signaling. Interestingly, these Foxp3^+^ DN cells were located in a transition state between DN1 and DN2 (CD4^-^CD8^-^CD3^-^TCR^-^CD44^high^CD25^low^). Our results indicate that Tcf7 is involved in preventing the premature expression of Foxp3 in DN thymocytes.

## Introduction

Regulatory T (T_reg_) cells are crucial to regulate immune responses, enforce immune homeostasis and maintain immune tolerance. Mutations in their lineage-defining transcription factor forkhead box P3 (Foxp3) cause severe autoimmune disease in human and mouse [1]. The thymus plays a critical role in immune tolerance through the deletion of auto-reactive T cells during negative selection and the generation of thymic-derived T_reg_ (tT_reg_) cells. T cell development from thymic precursor cells is a multi-step process [2]. At the CD4 and CD8 double-negative (DN) stage, T cell precursors start to rearrange their TCRβ gene. Upon successful expression of a pre-TCR, the precursors develop into double positive (DP) cells, which express both CD4 and CD8 co-receptors. At the DP stage, the TCRα chain is rearranged and the functional TCRαβ is expressed on the surface together with the CD3 complex. Selection into the T_reg_ lineage occurs during TCR-dependent positive and negative selection at the DP and CD4 single-positive (CD4SP) stage [3]. T_reg_ lineage commitment is favored in a TCR-affinity window that is heavily biased towards the affinity thresholds of negative selection [3]. Expression of Foxp3 occurs after T_reg_ lineage commitment and is a TCR- and cytokine-dependent process [3]. Foxp3 expression is detectable as early as the DP stage [4,5], but this is still a bit controversial and some authors favor the idea that Foxp3 expression does not occur before the CD4SP stage [6].

During T cell development in the thymus, transcription factor 7 (Tcf7), also known as T cell factor 1 (Tcf1), is involved at several stages [7,8]. Tcf7 is needed at the DN stage to ensure efficient T cell lineage commitment and T cell development is compromised at the DN and at the DN to DP transition state in Tcf7-deficient mice [9,10]. Survival of DP cells was also impaired in the absence of Tcf7 [11]. In addition, Tcf7 promotes the commitment of T cell precursors to the CD4 lineage [12].

We used a Tcf7-deficient mouse model to study the development of tT_reg_ cells. Interestingly, we detected the expression of the T_reg_ cell transcription factor Foxp3 in thymic DN cells under Tcf7-deficient conditions. These Foxp3^+^ DN cells were predominantly found in a DN1-DN2 transition state. This unexpected finding challenges the current understanding that Foxp3 expression can only occur after TCR signaling in thymocytes.

## Material and Methods

### Mice

Wild-type C57BL/6 (B6) mice were obtained from the Charles River Breeding Laboratories (Wilmington, MA, USA) or the Jackson Laboratory (Bar Harbor, ME, USA). *Tcf7*-deficient mice (Tcf7tm1Cle, ΔVII) were a gift from Hans Clevers (Hubrecht Institute, Utrecht, Netherlands) [9]. *Tcf7*-deficient mice (mixed 129-C57BL/6 background) were crossed to *Foxp3*-YFP (B6.129(Cg)-Foxp3tm4(YFP/cre)Ayr/J, Jackson number 016959) [13] or TEa (B6.Cg-Tg(Tcra,Tcrb)3Ayr/J, Jackson number 005655) [14] mice in our animal facility. For all experiments littermates were used as control animals. All mice were housed under specific-pathogen free conditions in the animal facility of the German Cancer Research Center.

### Ethics Statement

All animal experiments were reviewed and authorized by the local government (Regierungspräsidium Karlsruhe, Germany)

### Flow cytometry

Cells were labeled with fluorophore-conjugated antibodies and biotinylated antibodies were labeled with fluorophore-conjugated streptavidin (S1 Table). Intracellular staining of cells was performed using the intracellular Foxp3 staining buffer set (eBioscience). Cells were analyzed using the BD Bioscience Canto II or LSR II. Treestar FlowJo was used for the analysis of flow cytometry data.

### Statistical analysis

Statistical analysis of experimental data was performed using Prism software (GraphPad) or Excel (Microsoft). Employed statistical tests and corresponding parameters are mentioned in figure legends. Results were considered statistically significant if p-values were less than 0.05.

### Tissue isolation and sample preparation

For tissue isolation, organs were removed and processed by mashing and filtering to prepare single cell suspensions. Ammonium chloride—potassium bicarbonate lysis buffer was used to lyse erythrocytes in spleen samples.

### TEa antigen and genotyping PCR

The cognate antigen recognized by the TEa T cells is a peptide, called Eα, derived from the I-E molecule. The *I-E* gene in C57BL/6 mice contains an approximately 600bp deletion that encompasses this region and also prevents the generation of a protein product. PCR primers were designed to detect the presence or absence of this deletion. Primer sequences are listed in S1 Table. Surface expression of I-E could also be detected by staining with an antibody specific for I-Ek.

## Results and Discussion

### Expression of Foxp3 in DN thymocytes

**Fig 1:**
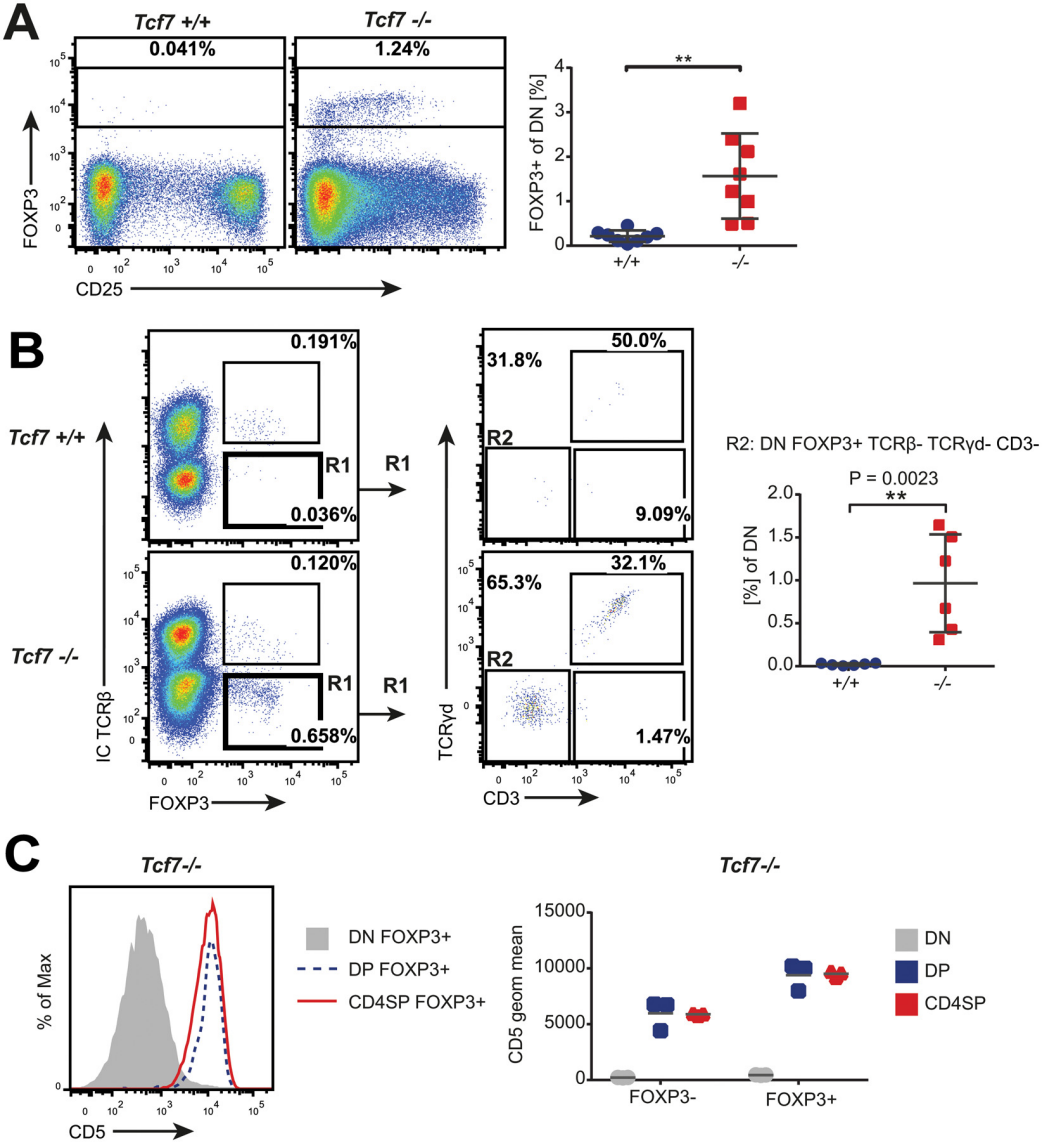
Foxp3 expression at the DN cell stage in Tcf7-deficient mice. (A) Representative plots and quantification of Foxp3 staining in CD4^-^CD8^-^ (DN) thymocytes from Tcf7^+/+^ and Tcf7^-/-^ mice (n = 8). (B) Left panels: Representative plots showing Foxp3 and intracellular (IC) TCRβ staining in DN thymocytes from Tcf7^+/+^ and Tcf7^-/-^ mice. Middle panels: TCRγδ and CD3 staining on DN Foxp3^+^TCRβ^-^ cells (gate R1). Right panel: Quantification of DN Foxp3^+^TCRβ^-^TCRγδ^-^CD3^-^ cells (gate R2) depicted as the percentage of total DN cells (n = 6). (C) Left panel: Representative histograms showing CD5 staining on Foxp3^+^ DN, Foxp3^+^ DP, and Foxp3^+^ CD4SP cells from Tcf7^-/-^ mice. Right panel: Quantification of CD5 geometric mean from DN, DP, and CD4SP Foxp3^+^ or Foxp3^-^ populations (n = 3). Mean + SD are shown for all quantified data. Numbers show percentages of cells within the indicated box. Each symbol represents an individual animal. ** P < 0.01 (unpaired t-test).

Based on the profound impact of Tcf7 on T cell lineage commitment [10], we decided to determine if Tcf7 also plays a role in tT_reg_ development. Therefore, we examined the DN cells for the presence of Foxp3. Surprisingly, we detected expression of Foxp3 in DN cells from Tcf7-deficient mice, representing almost two percent of total DN cells (Fig 1A). Results from anti-Foxp3 antibody staining were confirmed by crossing the Foxp3-YFP to the Tcf7-deficient strain (data not shown). Given the current understanding that Foxp3 expression identifies committed T_reg_ cells and, therefore, induction occurs after expression of a functional TCR, these results suggest that Tcf7 plays a role in inhibiting Foxp3 expression in DN cells.

To exclude the possibility that these CD4 and CD8 negative DN cells were actually more mature TCR-expressing thymocytes, we stained for CD3, TCRβ (including intracellular) and TCRγδ. Although some of the Foxp3^+^ DN cells found in the Tcf7-deficient mice expressed TCR proteins, about one percent of all DN cells expressed Foxp3 and had no evidence of CD3 or TCR expression (Fig 1B). This population was absent in Tcf7-proficient control mice (Fig 1B). In addition, the few Foxp3^+^ DN cells in Tcf7-proficient mice expressed TCRβ, suggesting that they were not true DN cells (Fig 1B—Left panels).

The absence of TCR expression did not prove that these Foxp3^+^ DN cells were true immature DN cells. In order to exclude that these cells had not previously received TCR signals, we examined them for CD5 expression. The trans-membrane glycoprotein CD5 is induced and stably expressed after positive selection and its expression level on the surface of thymocytes and mature T cells has been described to correlate with the overall avidity of the MHC-TCR interaction during positive selection [15]. Therefore, the presence of CD5 can be used as an indirect indication of previous TCR engagement. We tested the Foxp3^+^ DN cells from the Tcf7-deficient mice for CD5 expression and found that they did not express CD5, similar to Foxp3^-^ DN cells (Fig 1C). This is in contrast to all other Foxp3^+^ tT_reg_ populations and TCR-expressing T_conv_ cells in the thymus (Fig 1C), indicating that Foxp3^+^ DN cells had no previous TCR engagement and were distinct from all other Foxp3^+^ tT_reg_ cells. These results further showed that the absence of Tcf7 allows premature expression of Foxp3 in true DN cells.

### Foxp3-expressing DN cells do not respond to TCR stimulation *in vivo*

Since the discovery of Foxp3^+^ DN cells in Tcf7-deficient mice was so surprising, we decided to further examine their maturity in an *in vivo* model of negative selection. By forcing these cells to express a transgenic (Tg) TCR, we would determine if they have the downstream machinery necessary for productive TCR signaling. To this end, we chose a TCR-Tg model where the cognate antigen can be expressed on all thymic MHC class II positive antigen-presenting cells, including medullary thymic epithelial cells, cortical thymic epithelial cells and dendritic cells. We crossed the I-Ab-restricted CD4^+^ “TEa” TCR-Tg mouse line (C57BL/6 background) to the Tcf7-deficient mouse line (mixed C57BL/6 by 129 background) [14]. Following two rounds of mating, we identified Tcf7-deficient and Tcf7-proficient littermates that expressed the Tg TCR Vα2 and Vβ6. A fraction of the littermates also expressed the cognate antigen, which happens to be a peptide derived from the MHC class II I-E molecule expressed. In this model, the expression of the I-E molecule is contributed by the 129 of the mixed background (S1A and S1B Fig).

**Fig 2:**
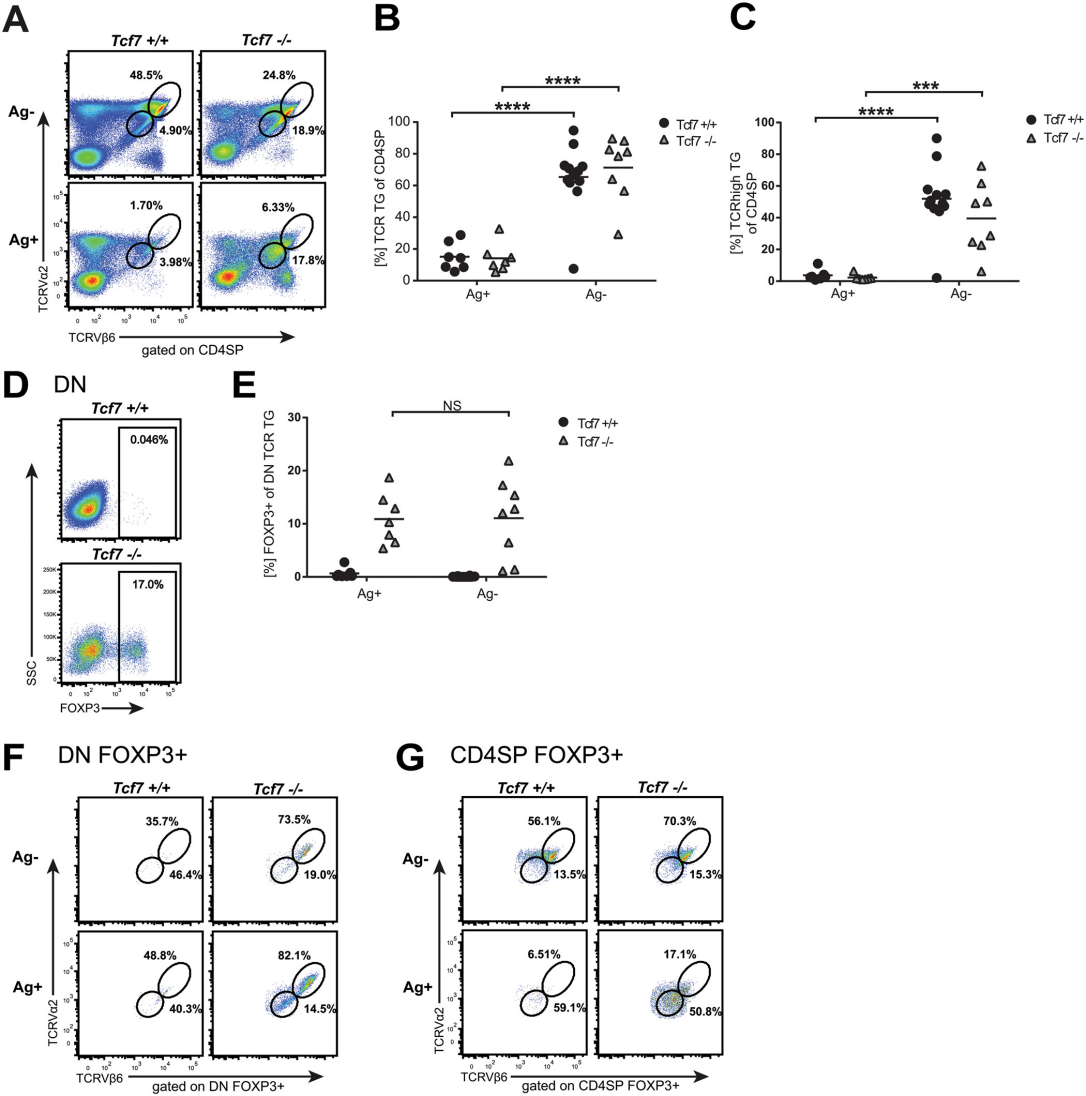
Analysis of Foxp3^+^ DN cells in TEa-Tcf7-deficient mice. (A) Representative plots showing TCRVβ6 and TCRVα2 expression on CD4SP thymocytes from TEa-Tcf7^+/+^ and TEa-Tcf7^-/-^ mice in the presence or absence of cognate antigen (Ag). The Tg TCR population is divided into TCR^high^ and TCR^low^ populations. (B-C) Quantification of the percentage of total (B) or TCR^high^ (C) TCRVβ6^+^TCRVα2^+^ cells among CD4SP thymocytes (n = 8). (D) Representative plots showing Foxp3 expression in DN TCRVβ6^+^TCRVα2^+^ thymocytes from TEa-Tcf7^+/+^ and TEa-Tcf7^-/-^ mice in the absence of Ag. (E) Quantification of Foxp3^+^ DN TCRVβ6^+^TCRVα2^+^ thymocytes from TEa-Tcf7^+/+^ and TEa-Tcf7^-/-^ mice in the presence or absence of Ag (n = 8). (F-G) Representative plots showing TCRVβ6 and TCRVα2 expression on DN Foxp3^+^ (F) or CD4SP Foxp3^+^ (G) thymocytes from TEa-Tcf7^+/+^ and TEa-Tcf7^-/-^ mice in the presence or absence of Ag. Cells are pre-gated on TCRVβ6^+^TCRVα2^+^. Each dot represents one individual animal and mean is shown for all quantified data. Numbers show percentages of cells within the indicated box. NS, not significant, *** P < 0.001, **** P < 0.0001 (unpaired t-test).

As expected, the presence of the cognate antigen resulted in the deletion of Tg TCR-expressing CD4SP T cells and the down-regulation of the Tg TCR in the surviving T cells (Fig 2A and 2B). For a more in-depth analysis, the Tg T cells were separated into TCR^low^ and TCR^high^ populations (Fig 2A). We found that in the presence of the antigen, the TCR^high^ Tg fraction of the CD4SP cells was severely reduced (Fig 2C). Similar findings were observed for the CD4SP Foxp3^+^ population (Fig 2G and S1C Fig). In this model, the antigenic stimulation was so profound that even CD4SP Foxp3^+^ Treg cells were deleted and surviving Foxp3^+^ Treg cells had lower expression of the Tg TCR (Fig 2G and S1C Fig). Importantly, no differences were observed between the Tcf7-proficient and Tcf7-deficient mice at the CD4SP stage in this model.

After having established a robust model with strong selection pressure, we analyzed the Foxp3^+^ DN population for the presence of a functional TCR signaling pathway. Flow cytometric analysis of the DN compartment revealed that Foxp3^+^ DN cells were again only found in the Tcf7-deficient mice, confirming our previous results (Fig 2D and 2E). Analysis of Tg TCR expression on Tg Foxp3^+^ DN cells revealed that Foxp3^+^ DN cells were mainly found in the TCR^high^ compartment (Fig 2F). Unlike the Foxp3^+^ CD4SP T cells, the Foxp3^+^ DN cells were not influenced by the presence of the cognate antigen. Expression levels of the Tg TCR and the frequency of Foxp3^+^ DN cells were not changed indicating that Foxp3^+^ DN cells did not functionally engage their TCR (Fig 2E–2G).

In this model, Foxp3^+^ DN cells from the Tcf7-deficient mice were forced to express a TCR. The absence of negative selection or down-regulation of TCR levels highlights their functional immaturity and adds additional support to the idea that these are true DN cells. These results further show that the Foxp3^+^ DN cells express Foxp3 in the absence of Tcf7. The absence of TCR-dependent reactivity of the Foxp3^+^ DN cells from the Tcf7-deficient mice is unlikely do to their different thymic localization. While CD4SP cells are located in the medulla, DN and DP cells are located in the cortex region of the thymus. However, negative selection can occur in both compartments [16] and our cognate antigen is ubiquitously expressed in all MHC-class II positive cells, including medullary thymic epithelial cells, cortical thymic epithelial cells, thymic dendritic cells, and thymic B cells.

### Foxp3+ DN cells are a transition state between DN1 and DN2

**Fig 3:**
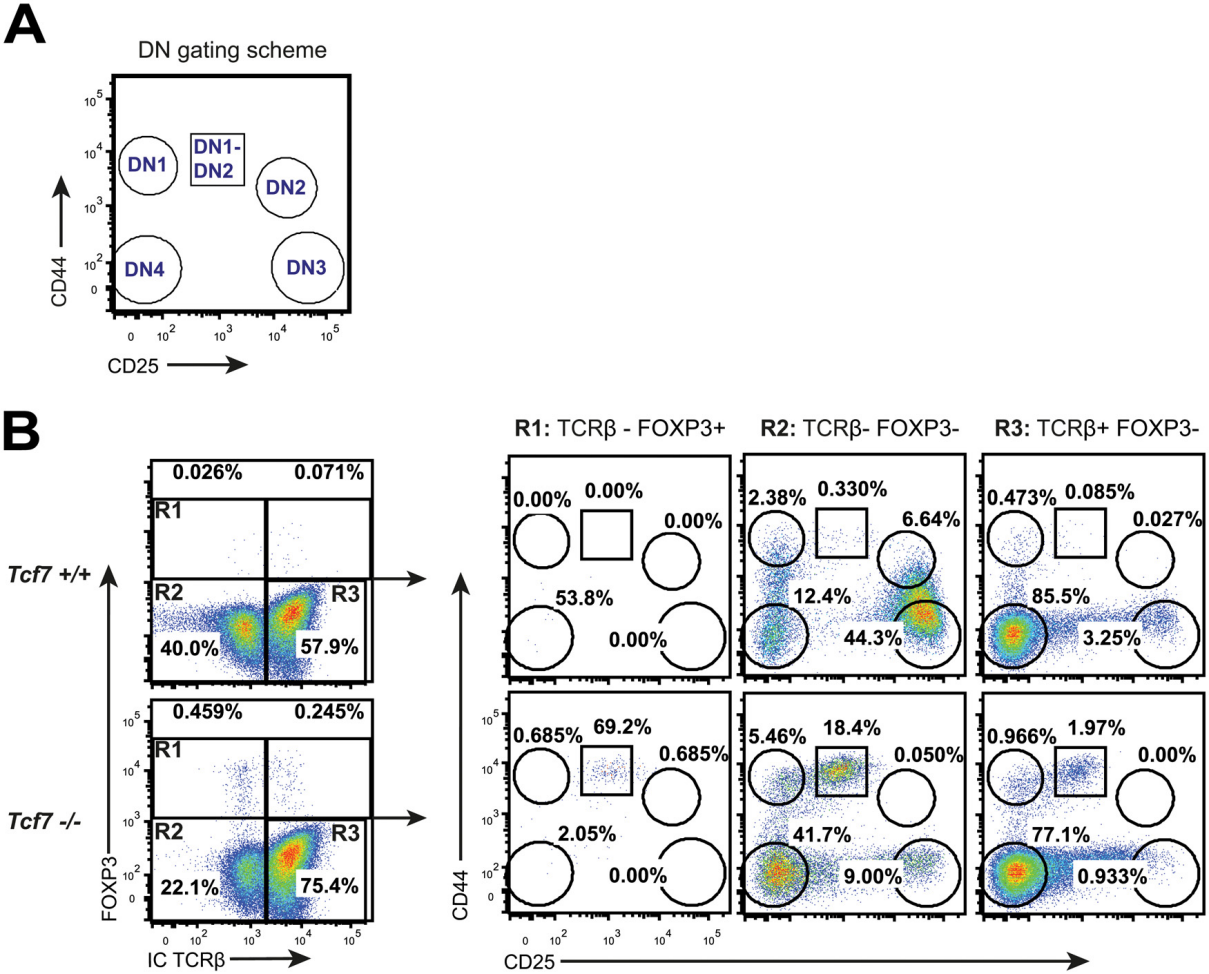
Expression of Foxp3 at thymic DN1-4 stages. (A-B) Flow cytometric analysis of CD4^-^CD8^-^ (DN) thymocytes from Tcf7^+/+^ and Tcf7^-/-^ mice. (A) Gating scheme for the identification of DN1-4 and DN1 to DN2 transition stages based on the expression of CD44 and CD25. (B). Left panels: Representative plots showing Foxp3 and intracellular (IC) TCRβ staining in DN thymocytes from Tcf7^+/+^ and Tcf7^-/-^ mice. Right panels: Representative plots showing CD44 and CD25 expression on the indicated DN populations (R1–R3). Numbers show percentages of cells within the indicated box.

To further characterize the Foxp3^+^ DN population in an attempt to identify how Tcf7 influences Foxp3 expression, we sub-divided the DN population into the DN1-DN4 developmental stages by the surface expression of CD44 and CD25 (Fig 3A). DN thymocyte development progresses from DN1 to DN4 with final TCRβ rearrangement taking place in DN3 [2]. Analysis showed that the Foxp3^+^ DN population in Tcf7-deficient mice was located in a transition state between DN1 and DN2, characterized by CD44^high^ and CD25^low^ expression (CD4^-^CD8^-^CD3^-^TCR^-^CD44^high^CD25^low^; Fig 3B—right panel R1). That positions the Foxp3^+^ DN population in a developmental stage that precedes TCRβ expression. In addition, this transition population was also highly enriched in the other DN populations in Tcf7-deficient mice (Fig 3B—right panel R2 and R3), while this DN1-DN2 population was barely present in Tcf7-proficient mice. Cells in this DN1-DN2 transition state (CD4^-^CD8^-^CD3^-^TCR^-^CD44^high^CD25^low^) have been previously described in the thymus of wild-type mice. They are more abundant in neonatal rather than in adult thymi. These cells are mostly T cell lineage committed and capable of generating all thymocyte sets (including DN3 and DN4, DP and SP). Additionally, they can migrate to the gut and locally differentiate into intraepithelial CD8αα T cells [17,18].

Tcf7 is highly expressed in the earliest thymic progenitors and it has been proposed that Notch signals induce Tcf7, and then Tcf7 in turn establishes the T-cell fate by up-regulating expression of T cell-essential genes such as *Gata3* and *Bcl11b* [10]. It is currently unclear if Tcf7 is directly inhibiting Foxp3 expression in DN cells or if the prolonged DN1-DN2 transition enables some DN cells to induce Foxp3 expression *de novo*. Foxp3 expression outside of the lymphocyte system has been suggested, although this is still controversial [19,20]. The appearance of Foxp3 expression in DN thymocytes before, and therefore independently of, TCR signaling is intriguing and may help to understand the signals that regulate Foxp3 induction. It is currently unclear if this Foxp3^+^ DN population has a physiological role. The fact that we cannot detect this population in wild-type mice does not preclude that they might exist under specific conditions and may represent a very early pre-commitment to the T_reg_ lineage.

## Conclusions

Investigation of the role of Tcf7 in the development of tT_reg_ cells, using a Tcf7-deficient model, lead us to the surprising finding that Foxp3 was expressed at the DN stage in the absence of Tcf7. Staining for TCR molecules (TCRβ, TCRγδ, CD3) together with staining for CD5 demonstrated that the expression of Foxp3 was independent of the presence of a TCR. We showed that most of these cells were enriched as CD4^-^CD8^-^CD3^-^TCR^-^CD44^high^CD25^low^ cells in a DN1-DN2 transition state. The generation of a TCR-Tg model, in which the Tg TCR was present in Tcf7-deficient Foxp3^+^ DN cells, showed that this population did not respond to TCR stimulation *in vivo*. Our results suggest that endogenous Tcf7 levels prevent the premature and TCR-independent expression of Foxp3 in DN cells. It also challenges the current understanding that Foxp3 expression can only occur after TCR signaling in thymocytes.

## Supporting Information

S1 Fig Identification of I-E^к^ antigen positive mice and expression of the transgenic TCR on CD4SP FOXP3^+^ cells. (A) Representative PCR from digested tail tissue from TEa-Tcf7^+/+^ and TEa-Tcf7^-/-^ mice. The cognate antigen (Ag) recognized by the TEa T cells is a peptide, called Eα, derived from the I-E molecule. The *I-E* gene in C57BL/6 mice contains an approximately 600bp deletion that encompasses this region and also prevents the generation of a protein product. PCR primers were designed to detect the presence (330bp fragment, Ag-, left lane) or absence of this deletion (957bp fragment, Ag+, right lane). (B) Representative plots showing I-E^к^ antigen expression in splenic CD19^+^ B cells. (C) Quantification of ratio of TCR^high^ to TCR^low^ Foxp3^+^ CD4SP TCRVβ6^+^TCRVα2^+^ thymocytes from TEa-Tcf7^+/+^ and TEa-Tcf7^-/-^ mice in the presence or absence of Ag as depicted in Fig 2G (n = 8). Each dot represents one individual animal and mean is shown for all quantified data. Numbers show percentages of cells within the indicated box. * P < 0.05, ** P < 0.01 (unpaired t test)
(EPS)

S1 Table Antibody and Primer list. (DOC)
