# Supplementary material for: Premature Expression of Foxp3 in Double-Negative Thymocytes
Source: PLoS One. 2015 May 15;10(5):e0127038. doi: 10.1371/journal.pone.0127038 (PMC4433242; doi:10.1371/journal.pone.0127038)
Supplement: S1 Table — (DOC) [file pone.0127038.s002.doc]

**S1 Table. Antibody and Primer list**

| **Antigen** | **Clone** | **Isotype** | **Supplier** |
| --- | --- | --- | --- |
| CD3ε | 145-2C11 | Arm. Hamster IgG | BioLegend |
| CD4 | RM4-5 | Rat IgG2a κ | BioLegend |
| CD4 | GK1.5 | Rat IgG2b κ | BioLegend |
| CD5 | 53-7.3 | Rat IgG2a κ | BioLegend |
| CD8a | 53-6.7 | Rat IgG2a κ | BioLegend |
| CD11b | M1/70 | Rat IgG2b κ | BioLegend |
| CD11c | N418 | Arm. Hamster IgG | BioLegend |
| CD19 | 6D5 | Rat IgG2a κ | BioLegend |
| CD25 | PC61 | Rat IgG1 λ | BioLegend |
| CD44 | IM7 | Rat IgG2b κ | BioLegend |
| I-E (MHC II) | 14-4-4S | Mouse IgG2a κ | BioLegend |
| TCR γ/δ | GL3 | Hamster IgG | BioLegend |
| TCR Vα2 | B20.1 | Rat IgG2a, λ | BioLegend |
| TCR Vβ6 | RR4-7 | Rat IgG2b, λ | BioLegend |
| TCR-β-chain | H57-597 | Arm. Hamster IgG | BioLegend |
| FoxP3 | FJK-16s | Rat IgG2a κ | eBioscience |
| Biotin |  | Streptavidin | BioLegend |
| Fixable Viability Dye |  |  | eBioscience |
|  |  |  |  |
|  |  |  |  |
| **Primer for IEk genotyping** | |  |  |
| IEk ForP | CTAGCCCACTGCAAAAGGAG | |  |
| IEk RevP | CCCAGGAATGAAACTGGTTG | |  |

.
